# Emotional valence boosts partial and specific source memory

**DOI:** 10.3758/s13423-026-02888-6

**Published:** 2026-04-02

**Authors:** Nikoletta Symeonidou, Maria Lee, Beatrice G. Kuhlmann

**Affiliations:** Department of Psychology, School of Social Sciences, University of Mannheim, 68161 Mannheim, Germany

**Keywords:** Specific source memory, Partial source memory, Emotion-enhanced memory, Multinomial modeling

## Abstract

Previous research has shown that source memory is enhanced for emotionally valenced sources. However, it remains unclear whether this enhancement relies on better memory for specific source details or merely improved memory for the source-valence category. The present study addresses this question and disentangles emotion-based effects on partial and specific source memory using multinomial modeling. Participants (*N* =67) encoded neutral words paired with positive, negative, or neutral source images with three similar images per valence (all matched on arousal). At test, participants were presented with all nine source images and, for each test word, identified the specific corresponding source image or indicated that the word was new. Results revealed that both partial (valence category) and specific (particular image) source memory were superior for emotionally valenced sources compared to neutral ones. Further notable, specific source memory was higher for positive than for negative sources, indicating a positivity effect. These findings extend prior research by demonstrating that source emotionality not only enhances the recollection of partial source information but also of specific source details. The observed positivity effect is further discussed in terms of a potentially greater associative potential of positive stimuli, and methodological considerations for future research are outlined.

**Supplementary Information:**

The online version contains supplementary material available at 10.3758/s13423-026-02888-6.

It is well established that memory is often better for emotional compared to neutral stimuli—a phenomenon known as the emotion-enhanced memory (EEM) effect. This effect has been robustly demonstrated in item memory, meaning that when a central to-be-encoded item—such as a word or image—is emotional, its subsequent recognition or recall is enhanced (Gao et al., 2024; Mather, 2007; Talmi, 2013). There is accumulating evidence that EEM effects similarly occur in source memory—that is, memory for the contextual details associated with a central item (Johnson et al., 1993). Focusing primarily on emotionally valenced sources, this line of research has shown that when a neutral item (e.g., word) is encoded with a positive or negative as opposed to neutral source (e.g., background image), memory for the emotional source is enhanced (e.g., Bell & Buchner, 2010; Smith et al., 2005; Symeonidou & Kuhlmann, 2024).^1^

However, a crucial limitation of most of these studies is that they assess source memory at the level of general valence categories. That is, during the test phase, participants are typically asked to indicate whether an item was previously paired with a positive, negative, or neutral source or to identify it as new (see, e.g., Smith et al., 2005; Symeonidou & Kuhlmann, 2024). In such test designs, merely remembering the general emotional valence of the source, rather than its specific details, is sufficient for a correct source judgment. Although source memory has often been linked to the process of recollection—that is, the conscious retrieval of specific contextual details associated with an event (Johnson et al., 1993)—previous research has demonstrated that accurate source judgments can occur in the absence of full vivid recollective experiences (Hicks et al., 2002). Such findings led to the proposal of *partial* source memory, motivating the development of statistical models that disentangle partial from *specific* (fully detailed) source memory (Dodson et al., 1998; Klauer & Wegener, 1998). This raises an important question: Does the observed source-memory enhancement for emotional sources reflect a more detailed recollection of specific source features, or does it stem from better memory for the general valence category? The present study addresses this question by analyzing emotional valence effects on source memory with a model that distinguishes partial and specific source memory.

As mentioned, research on emotional sources remains relatively limited. The most systematic evidence stems from studies manipulating source valence through normed or pre-rated emotional stimuli (e.g., Bell & Buchner, 2010), with some studies additionally controlling for arousal (e.g., Symeonidou & Kuhlmann, 2024). This line of research has quite consistently demonstrated enhanced source memory for emotionally valenced sources provided that participants’ attention was directed towards the emotional value of the source context at encoding (e.g., through pleasantness judgments). Importantly, however, in most of these studies, the memory tests did not require recollection of specific source details to make a correct source judgment. For example, in Symeonidou and Kuhlmann (2024), source memory was assessed using a four-alternative forced-choice format: Participants first encoded neutral word items on either a positive, neutral, or negative source image and later, at test, chose for each word (previously presented or new) between three source images (one negative, one neutral, one positive), or the option “new.” In this test format, recalling only the general valence of the associated source (e.g., item was paired with “something pleasant” vs. “something unpleasant”) would be sufficient for a correct source response, without recollecting specific details of the source image. Consequently, as already discussed by Smith and colleagues (2005), such test designs confound partial and specific source memory, as accurate responses can either reflect memory for the general source-valence category or memory for the specific source image. While research on emotional *item* memory has provided robust evidence that emotion—particularly high negative arousal—enhances memory for both the gist and the specific details of emotional items (e.g., Kensinger et al., 2007; Mather, 2007), comparable systematic research differentiating memory for gist versus details for emotional sources is lacking.

To our knowledge, only two studies investigated such effects for emotional sources, both conducted by Bell and colleagues. In Bell, Giang, and Buchner (2012), participants encoded neutral faces as items, each paired with unique behavioral descriptions functioning as sources. These descriptions were either neutral or negative, with the negative category further divided into two types (cheating or disgusting behavior). Results indicated that participants not only showed enhanced memory for the negative versus neutral behaviors but were also able to distinguish between the two negative source types (cheating vs. disgusting behaviors). However, the authors later acknowledged that this finding might reflect source memory at the level of the behavioral category, rather than memory for specific source details (i.e., remembering the specific disgusting or specific cheating behaviors) and therefore conducted a second study (Bell, Buchner, et al., 2012), again pairing neutral faces with either cheating, trustworthy, or irrelevant behavioral descriptions. This time, however, source memory was first assessed via a standard four-alternative forced-choice task targeting the behavioral category (partial source memory), followed by a cued-recall test assessing memory for the specific behavioral description (specific source memory). While results revealed enhanced memory for the cheating (i.e., negative) versus trustworthy and irrelevant source-category, no corresponding enhancement was observed for specific source details, suggesting that EEM effects were limited to partial source memory.

However, several limitations temper these findings. In addition to broader methodological concerns such as the lack of arousal control (cf. Symeonidou & Kuhlmann, 2024) and the use of semantically emotional (i.e., affective meaning conveyed via stimulus’ semantic content, as in behavioral descriptions) rather than directly emotional source stimuli (i.e., affect conveyed immediately by the stimulus itself, as in images; cf. Bayer & Schacht, 2014), the two memory tests employed in Bell, Buchner, et al. (2012) differed substantially in retrieval demands. While partial source memory was assessed using a forced-choice test, specific source memory relied on a more effortful recall procedure. Thus, participants may have encoded specific source details but failed to retrieve them in the recall task. Indeed, in other studies comparing partial versus specific source memory (e.g., Klauer & Wegener, 1998), all specific source options were presented at test such that participants needed to recognize (instead of recall) the correct specific source, thereby minimizing retrieval demands. Building on this body of research, and to address these limitations, the current study employed normed background images as perceptually emotional sources, with all source exemplars presented in the test phase, facilitating retrieval (see Method section for details). Crucially, there were multiple images (exemplars) per valence category. Like Bell, Buchner, et al. (2012), we used the *Partial-Source Multinomial Processing Tree Model* originally proposed by Dodson et al. (1998), which models test responses as sequence of latent cognitive processes. Specifically, the model (see Method for details) assumes that after successful item recognition, participants may also recollect the specific source image, which leads to a correct source response. If specific source memory fails, participants may still remember sources’ valence (partial source memory), resulting in a correct valence-category response. If memory fails entirely, responses are assumed to reflect guessing processes.

Concerning our hypotheses—given prior evidence showing enhanced source memory for emotionally valenced contexts—we expected that this effect manifests at least in partial source memory (i.e., memory for the general source-valence category). With respect to specific source memory, we deemed two patterns as possible: Either (a) there is no EEM effect in specific source memory, implying that previously reported effects primarily reflect memory for source valence, or (b) EEM effects extend to specific source memory, indicating that emotion also benefits recollection of specific source details.

## Method

**Fig. 1:**
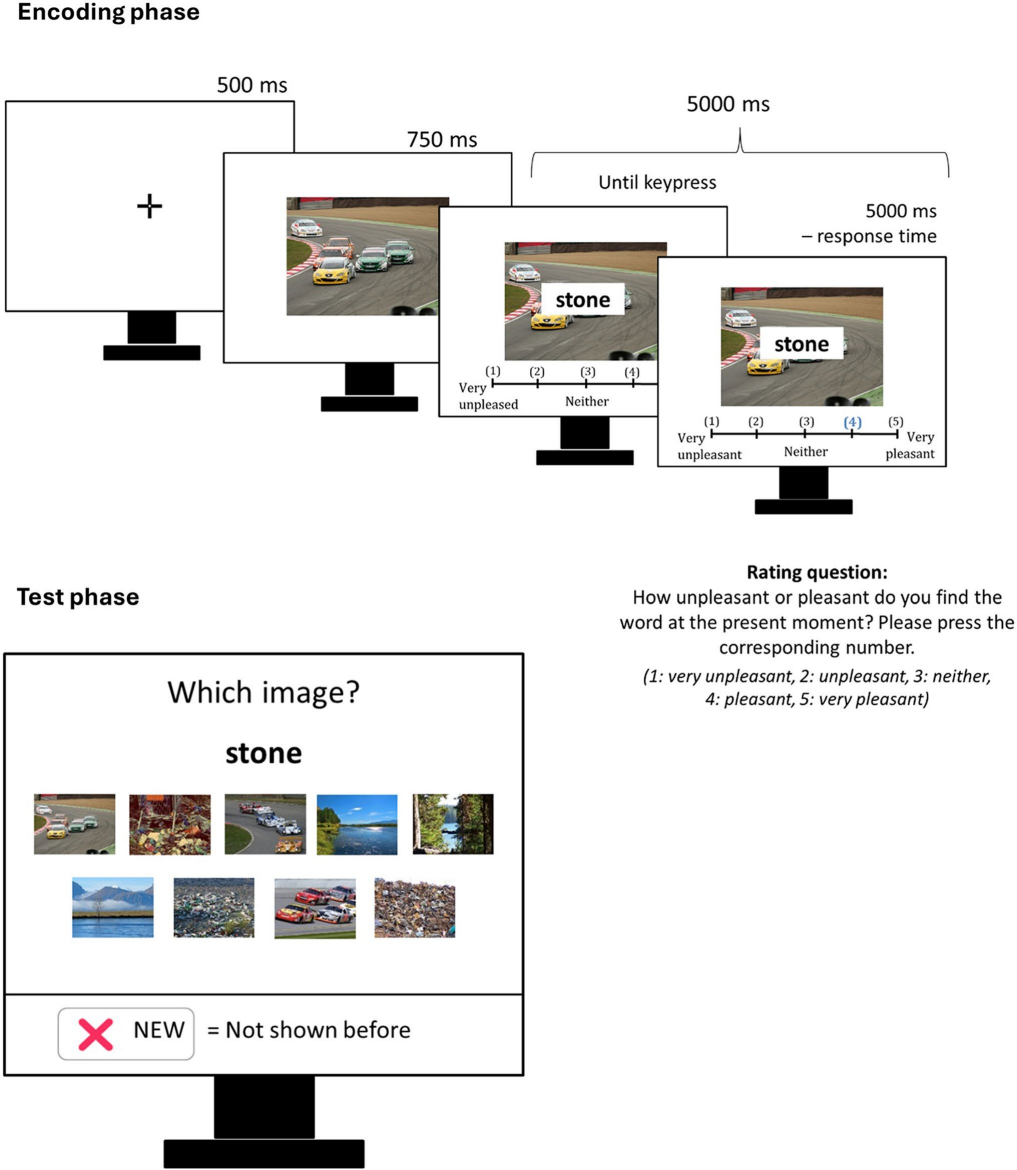
Illustration of the experimental flow (encoding & test phase). The encoding and test phase were separated by a three-minute interval, which was filled with a pattern-comparison task (not shown here). The full-resolution images are freely available under https://www.benedekkurdi.com/%23oasis. The nine images used in this study can be retrieved based on the original file names provided in Table 1.

The study closely followed the methodological approach in Symeonidou and Kuhlmann (2024). That is, at encoding, participants provided pleasantness ratings for neutral word items, which were superimposed on a negative, neutral, or positive background image. In a subsequent source-monitoring test, they were asked to recognize the previously presented word items along with their associated source images (Fig. 1). The critical methodological deviation in the present study was that multiple source images per valence category were used, rather than a single image. This enabled the differentiation between partial source memory (i.e., memory for the emotional valence category of the source) and specific source memory (i.e., memory for the exact source image).

### Design

A one-factorial design with source valence (negative, neutral, positive source images) as a within-subjects factor was employed. The primary dependent variables were partial and specific source memory, quantified via the *P* parameter and the *d* parameter of the Partial-Source Model (Dodson et al., 1998; as implemented in Klauer & Wegener, 1998), respectively.

### Participants

The sample primarily consisted of students from the University of Mannheim. Sample size was determined based on Experiment 1 by Symeonidou and Kuhlmann (2024), who employed a similar procedure and found a reliable source-memory benefit for the emotional source categories (akin to partial source memory) with *N* = 68. We could not conduct a properly informed a priori power analysis because no prior study had applied a comparable paradigm using the Partial-Source Model to disentangle partial and specific source memory. As such, making reasonable specific assumptions on parameter values, which is necessary to conduct an a priori power analyses with MPT models, was not feasible. Instead, we conducted a post hoc power analysis (detailed in the online supplement, section Supplemental Analysis 1), which revealed a power of 1 − β ≥ .85 to detect parameter differences of .15 between emotional and neutral source memory parameters in both partial and specific source memory. Note that this parameter difference represents a conservative estimate of EEM effects in source memory, as prior research typically reported differences larger than .20 (e.g., Bell, Buchner, et al., 2012; Symeonidou & Kuhlmann, 2024). Therefore, with *N* = 67, our study had sufficiently high power to detect small valence effects in both source memory parameters.

Of the 77 participants initially recruited online, ten were excluded according to predefined criteria. Specifically, three participants were excluded because they never used the “new” option in the test, three were excluded due to more than 15% missing responses during the encoding task, one participant was excluded for repeated participation, and three were excluded for not fulfilling demographic or health-related inclusion criteria (age range between 18 and 35 years; German as native language [i.e., learned before the age of 6]; and no diagnoses of depression, anxiety disorders, or posttraumatic stress disorder within the past 6 months).The final sample consisted of *N* = 67 participants (56 women, 11 men), with a mean age of 23.18 years (*SD* = 4.31).

### Material

#### Source images

For each valence category (negative, neutral, and positive), three distinct source images were selected, resulting in a total of nine images. More specifically, the negative category consisted of three different images of garbage, the positive category of three different lake images and the neutral category of three car race images (see Fig. 1, test phase). The images were all drawn from the OASIS image database (Kurdi et al., 2017) and the category labels were adopted from there.

Image selection was primarily guided by clear-cut valence and arousal criteria: the positive and negative image categories were matched for absolute valence (i.e., equal distance from the scale midpoint); additionally, all image categories had matched, medium arousal levels (see Table 1). From the image categories meeting these valence and arousal criteria, we selected three categories with three exemplars each based on two key similarity metrics, namely *entropy* (Machado et al., 2015), indexing image complexity, and the *Structural Similarity Index* (SSIM; Brunet et al., 2012), measuring image contrast, brightness, and structural features. More specifically, because the OASIS database does not provide entropy and SSIM values, we first computed these metrics using Python notebooks within the AI-based Google Colab environment (Google, 2024). Then, within the described valence and arousal constraints, we let the AI select triplets per category such that mean entropy and SSIM were comparable across categories while maintaining at least some variance within each category (see Table 1). This was intended to avoid excessive similarity among images within a category, which could overly impair specific source memory. The link to the Google Colab script is provided in the *Open Practices Statement* section.

**Table 1:** Mean ratings of the selected source images

Valence category	Original file name (& label in the model)	Valence	Absolute valence	Arousal	SSIM (AI- calculated)	Entropy (AI- calculated)
Positive	Lake 1.jpg *(Lake-1)*	6.25 (0.90)	2.25 (0.90)	3.97 (1.96)	0.06	0.16
Positive	Lake 2.jpg *(Lake-2)*	6.39 (0.87)	2.39 (0.87)	3.89 (2.02)	0.17	0.02
Positive	Lake 5.jpg *(Lake-3)*	5.95 (1.06)	1.95 (1.06)	4.30 (1.88)	0.17	0.00
Neutral	Car race 2.jpg *(Car-race-1)*	4.37 (1.07)	0.37 (1.07)	3.59 (1.81)	0.23	0.02
Neutral	Car race 3.jpg *(Car-race-2)*	4.40 (1.08)	0.40 (1.08)	3.69 (1.80)	0.22	0.01
Neutral	Car race 4.jpg *(Car-race-3)*	4.15 (1.14)	0.15 (1.14)	3.48 (1.73)	0.19	0.09
Negative	Garbage dump 2.jpg *(Garbage-1)*	1.60 (0.78)	2.4 (0.78)	3.78 (1.96)	0.02	0.02
Negative	Garbage dump 3.jpg *(Garbage-2)*	2.00 (1.02)	2.00 (1.02)	3.47 (1.80)	0.02	0.35
Negative	Garbage dump 4.jpg *(Garbage-3)*	1.64 (0.81)	2.36 (0.81)	3.79 (1.95)	0.02	0.01

Images were drawn from the Open Affective Standardized Image Set (OASIS; Kurdi et al., 2017). The labels in the brackets correspond to the labels used in the multinomial model. The full-resolution images are freely available (https://www.benedekkurdi.com/%23oasis) and can be retrieved based on the provided original file names. Mean valence and arousal ratings (standard deviation in brackets) were drawn from the original OASIS norms. SSIM (= Structural Similarity Index) and entropy were calculated using Python notebooks within the Google Colab environment (Google, 2024). Images per category were selected such that on average negative and positive sets matched on absolute valence, and all three sets matched on arousal.

#### Word items

The same word stimuli as in Symeonidou and Kuhlmann (2024) were employed in the present study. Specifically, 60 neutral words from the Berlin Affective Word List Reloaded (BAWL-R; Võ et al., 2009) were used. These words were divided into four lists of 15 items each, carefully matched on average neutral valence ([− 1.5; 1.5] on a rating scale ranging from − 3 [negative] to + 3 [positive]), low arousal (≤ 2.5 on a 5-point rating scale with higher values indicating higher arousal levels), and moderate imageability (> 3 on a 7-point rating scale with higher values indicating higher imageability). For each participant anew, the lists were randomly assigned to be paired with either negative, neutral, or positive source images, while one list served as distractor items in the test phase.

### Procedure

The study was programmed using lab.js (Henninger et al., 2022) and conducted online via the OpenLab server platform (Shevchenko, 2022). Participants first provided informed consent and completed a set of questions to determine eligibility for participation (see above). Eligible participants then completed a scaling task to verify that their screen met the minimum size requirement (i.e., browser window height of at least 4.72 inches) and to adjust the source images to their screen size. Next, participants entered the encoding phase (see Fig. 1), in which they were instructed to rate overall 45 neutral words on a 5-point Likert scale (ranging from 1 = *very unpleasant* to 5 = *very pleasant*) based on how each word made them feel at that moment. Each encoding trial began with a 500-ms fixation cross, followed by the presentation of one of the nine source images—drawn from the three valence categories (positive, neutral, negative)—for 750 ms. Subsequently, a neutral word appeared in a white box overlaid on the image and the 5-point rating scale was simultaneously displayed beneath the word–image pair. Participants had a total of 5 s to provide their rating. If a response was given before the time limit, the selected number turned blue to indicate the response was logged, and the word–image pair remained on screen until the full 5 s had elapsed. After excluding three participants missing more than five trials (i.e., > 15%; see above), the vast majority of participants (60 out of 67) missed only two or less trials and only one person missed the maximum of five trials. The mean response time for the pleasantness ratings was 2,212 ms (with a between-participants variation of *SD* = 970 ms). The 45 neutral words were evenly distributed across the nine source images (i.e., five words per image) and presented in randomized order, with a maximum of four consecutive repetitions of the same image allowed. Immediately after the encoding phase, participants completed three blocks of a pattern comparison task (Salthouse, 1996) to reduce potential recency effects. In the subsequent self-paced test phase, participants were shown all 45 previously studied words along with 15 new distractor words, presented individually. Each test word appeared alongside all nine source images, with their screen position randomized per participant but remaining constant across all trials for that individual. Participants were asked to select via mouse click the image with which the word had been paired during encoding, or to choose the “new” option if they believed the word had not been presented before. The “new” option was consistently displayed as a button in the footer of the screen (Fig. 1). Accordingly, partial source memory, specific source memory, and item memory were assessed simultaneously (cf. original procedure in Dodson et al., 1998; Klauer & Wegener, 1998). Following the test phase, participants were asked whether they had any issues with the presentation of text or images and whether they utilized any assistance during the task. There was also a field for open feedback. No major issues were reported. Participants were then debriefed about the research aim and redirected to another website (SoSci Survey; Leiner, 2024) to anonymously get course credit for their participation.

### Multinomial model-based analysis for partial and specific source memory

To analyze source memory responses, we employed the Partial-Source Model introduced by Dodson et al. (1998); however, implementing the two-high-threshold (instead of the original one-high-threshold) assumption for item recognition, as suggested by Klauer and Wegener (1998). The two-high-threshold assumption has been validated in earlier work (Bayen et al., 1996) and is implemented in the classical two-high-threshold model of source monitoring (2HTSM), which is often used for data analysis in standard, simpler source-monitoring designs (see Kuhlmann et al., 2021, for a review). The assumption is thus deemed to more appropriately reflect the item-recognition processes involved in source-monitoring decisions. Further, as the original Partial-Source Model was formulated for two source categories with two specific exemplars each, we extended the structure to accommodate three source valence categories (negative, neutral, positive) with three source exemplars per category**,** following the logic outlined in Keefe et al. (2002), who extended the 2HTSM from two to three sources.^2^ The structure for the guessing paths was also adapted from Klauer and Wegener (1998).

**Fig. 2:**
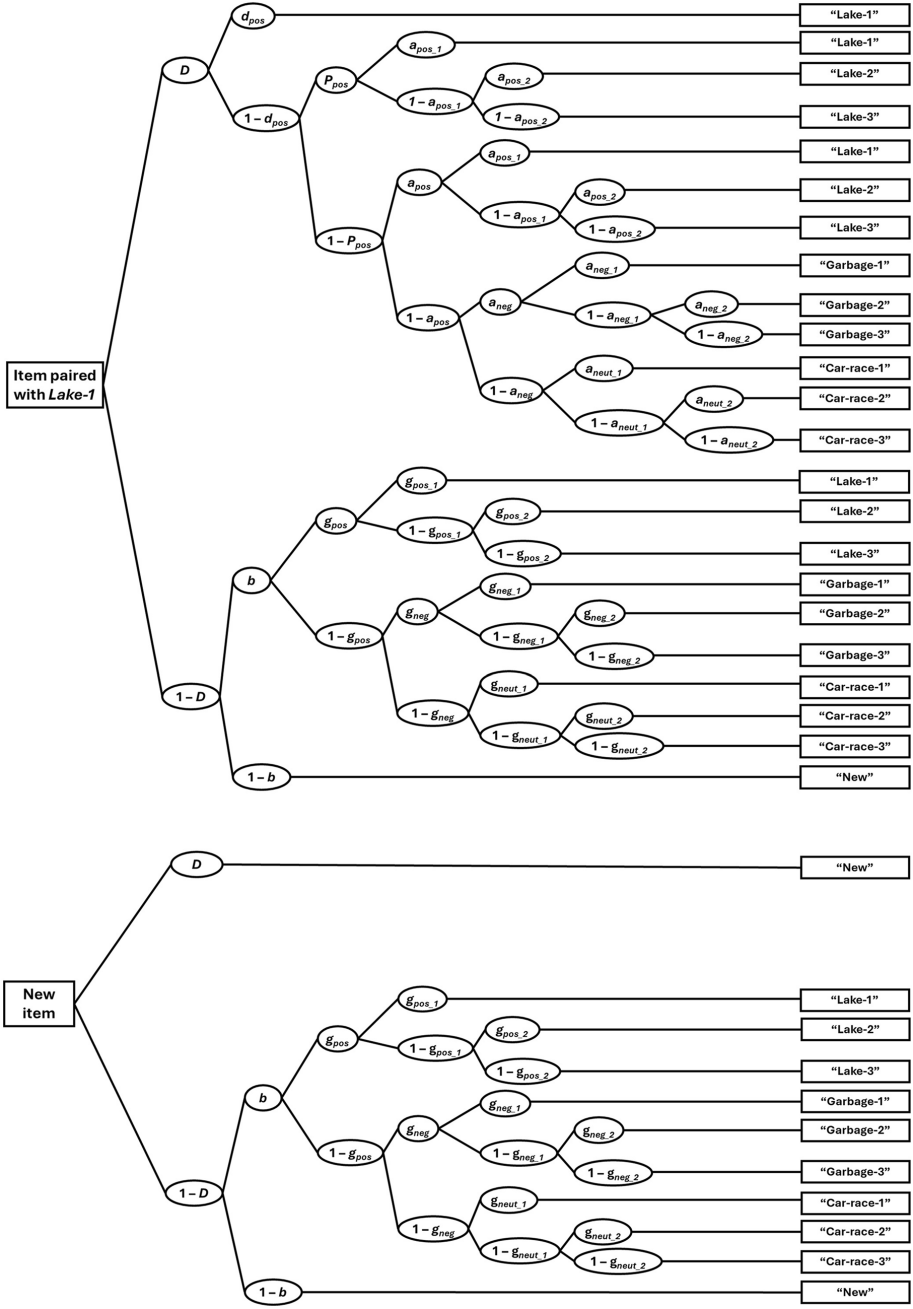
Depicted is the two-high-threshold version of the Partial-Source Multinomial Model (Dodson et al., 1998; Klauer & Wegener, 1998), exemplified for items originally paired with the *Lake-1* source image (upper tree) and new items (lower tree). *i* denotes the original source-valence category (in this example: positive) with *i* ∈ {negative, neutral, positive}. Boxes on the right represent participants’ responses in the source-memory test. *D* = item recognition; *d*_*i*_ = specific source memory for the source exemplar; *P*_*i*_ = partial source memory for the source-valence category; *b* = guessing that a word was previously presented; *a*_*pos*_ and *a*_*neg*_ = probability of guessing the positive and negative source-valence category, respectively, for recognized word items (guessing the neutral category is modeled via 1-*a*_*neg*_); *a*_*pos_1*_, *a*_*pos_2*_, *a*_*neg_1*_, *a*_*neg_2*_ , *a*_*neut_1*_, and *a*_*neut_2*_ = probability of guessing the specific source image (exemplar 1 or 2) of the respective source-valence category (positive, negative, neutral) for recognized word items (guessing exemplar 3 is modeled via the complementary probability, e.g., 1-*a*_*pos_2*_ for the *Lake-3* exemplar). The *g* parameters represent similar processes as the respective a parameters but for unrecognized items (i.e., 1-*D)*. Please refer to the main text for more details on the model.

Our model variant is illustrated in Fig. 2, showing the sequence of assumed underlying processes on the left side, linked to the observed response categories on the right side—exemplified for items originally paired with the *Lake-1* source image. Notably, for coding the response categories, it is important to differentiate between correct category responses and correct image responses. For instance, if a word was originally paired with the *Lake-1* image and the participant chose the *Lake-2* image in the memory test, this counts as correct category response even though the specific source image was incorrect.

The model begins with parameter *D*, which reflects item recognition—that is, recognizing a word as old or identifying it as new. If a word is recognized, the specific source image may be retrieved with probability *d*_*i*_, reflecting specific source memory; this parameter was allowed to vary across the three valence categories (with *i* indexing positive, neutral, and negative source valence). Crucially, if the specific source image cannot be retrieved, participants may still remember its valence with probability *P*_*i*_, resulting in a correct valence-category response. Thus, *P*_*i*_ represents partial source memory and can also differ across valence categories. Participants then need to guess the specific image, which, for the positive images, is modeled with probability *a*_*pos_1*_, *a*_*pos_2*_, and 1-*a*_*pos_2*_, for the *Lake-1*, *Lake-2*, or *Lake-3* image, respectively. A similar logic applies to the negative and neutral images, which are accordingly modeled with *a*_*neg_1*_, *a*_*neg_2*_, 1 − *a*_*neg_2*_, for guessing the (negative) *Garbage-1*, *Garbage-2,* or *Garbage-3* image*; *and* a*_*neut_1*_, *a*_*neut_2*_, 1 − *a*_*neut_2*_, for guessing the (neutral) *Car-race-1*, *Car-race-2,* or *Car-race-3* image, respectively. Note that *P* and *d* were our parameters of interest and allowed testing whether specific and partial source memory is enhanced for emotionally valenced sources. If both specific and partial source memory fail, participants source responses are entirely based on guessing. The process of guessing the source-valence category is modeled via probabilities *a*_*pos*_, *a*_*neg*_, and 1 − *a*_*neg*_, for guessing the positive, negative, or neutral source-valence category, respectively. Conditional on the guessed valence category, the specific source image is then guessed as described above. For example, within the positive source category, guessing the *Lake-1*, *Lake-2*, or *Lake-3* image is modeled by the parameters *a*_*pos_1*_, *a*_*pos_2*_, and 1 − *a*_*pos_2*_. Of note, this specification implies that, whenever specific source memory fails, the probability of selecting a particular source image is the same regardless of whether partial source memory is available. If not only source memory but also item memory fails (1 − *D*), all test responses are driven by guessing processes. The probability of guessing that a test word is old is modeled by parameter *b*. Conditional on guessing “old”, the model specifies guessing at the level of the source-valence category via parameters *g*_*pos*_, *g*_*neg*_, and 1-* g*_*neg*_, for the positive, negative, and neutral valence, respectively. Given a guessed source-valence category, the specific source image is then guessed. For example, within the positive category, guessing the *Lake-1*, *Lake-2*, or *Lake-3* image is modeled by *g*_*pos_1*_, *g*_*pos_2*_, and 1 − *g*_*pos_2*_. A similar logic applies to the negative and neutral images, which are modeled with *g*_*neg_1*_, *g*_*neg_2*_, 1 − *g*_*neg_2*_, for guessing the (negative) *Garbage-1*, *Garbage-2,* or *Garbage-3* image*;* and *g*_*neut_1*_, *g*_*neut_2*_, 1 − *g*_*neut_2*_, for guessing the (neutral) *Car-race-1*, *Car-race-2,* or *Car-race-3* image*,* respectively. In short, the *g* parameters represent similar processes as the *a* parameters but for unrecognized items.

Notably, the depicted model in Fig. 2 represents a constrained version which we specified based on theoretical considerations and prior empirical findings. More specifically, we implemented the following parameter restrictions: Parameter *D* was restricted to be equal across all source images and equal to distractor detection. This reflects the assumption that word recognition should not differ depending on the accompanying background image (see also Bell, Buchner, et al., 2012; Symeonidou & Kuhlmann, 2024). Parameter *d*_*i*_ was allowed to vary across valence categories but was constrained to be equal across the three source exemplars within each valence category (e.g., the *Lake-1* image should be equally memorable as the *Lake-2* and *Lake-3* image). The same restriction was applied to partial source memory *P*_*i*_. Although unintuitive at first glance, *P*_*i*_ could, in principle, differ across the three source images of a given valence category (cf. Dodson et al., 1998, Appendix D). For example, if one positive image is experienced as more positive than the others, partial source memory for the positive valence might be higher for that exemplar. However, because we had carefully matched the three images within each valence category in terms of valence and arousal, such differences in memorability were unlikely for both, the valence category (*P*) and the specific picture (*d*), justifying these restrictions. Finally, as already described before, the probability for guessing a specific source image (*a*_*ij*_) was the same regardless of whether partial source memory was available or not.

Parameter estimation was based on the aggregated observed response frequencies and model fit was assessed with maximum likelihood (ML) estimation methods, using the software MultiTree (Moshagen, 2010). To foreshadow, the specified base model fitted the data, implying that the above-described restrictions were in line with the observed response frequencies.

To evaluate differences between model parameters—and thereby test our hypotheses—we conducted model fit comparisons using parameter restrictions. For instance, to assess whether specific source memory was enhanced for emotionally valenced sources compared to neutral ones, the three *d* parameters were constrained to be equal. A significant decline in model fit following this restriction would indicate that the parameters differ meaningfully (see *Results* section).

Further note that, in section Supplemental Analysis 2 of the online supplement, we also report analyses with (average) conditional source identification measures ([A]CSIMs), which are traditionally used as empirical measure for source-memory performance (Bröder & Meiser, 2007; Murnane & Bayen, 1996). These analyses yielded descriptively very similar results to the model-based approach, supporting the overall robustness of the findings. Of note, however, (A)CSIM scores do not account for systematic guessing biases and therefore provide only a suboptimal measure of participants’ partial and specific source memory (Murnane & Bayen, 1996). Thus, we argue that the model-based analysis presented in the following offers a more appropriate approach to the data.

## Results

Alpha level was set to α = .05 for all analyses.

### Pleasantness ratings

A within-subject ANOVA with Greenhouse–Geisser correction tested how source valence influenced participants’ pleasantness ratings for the neutral word items. A main effect of source emotionality occurred, *F*(1.52, 100.54) = 62.06, *p* < .001, η_p_^2^ = .49. Following up on this main effect, Bonferroni–Holm adjusted pairwise comparisons showed that, as expected, words overlaid on positive source images were rated as more pleasant (*M* = 3.31, *SD* = 0.49) compared with those on neutral (*M* = 2.71, *SD* = 0.46), *t*(66) = 8.26,* p* < .001, *d* = 0.90, and those on negative images (*M* = 2.42, *SD* = 0.62), *t*(66) = 8.76, *p* < .001, *d* = 1.33. Similarly, words on neutral images were rated as more pleasant than words on negative images, *t*(66) = 4.40, *p* < .001, *d* = 0.43. This suggests that the sources’ valence influenced participants’ rating of the neutral items, replicating previous research (Bell, Buchner, et al., 2012; Symeonidou & Kuhlmann, 2024).

### Source memory

The specified base model fitted the data, *G*^2^(66) = 82.00, *p* = .088. Resulting estimates for all parameters of the model are reported in Supplemental Table S1.

#### Partial source memory

**Fig. 3:**
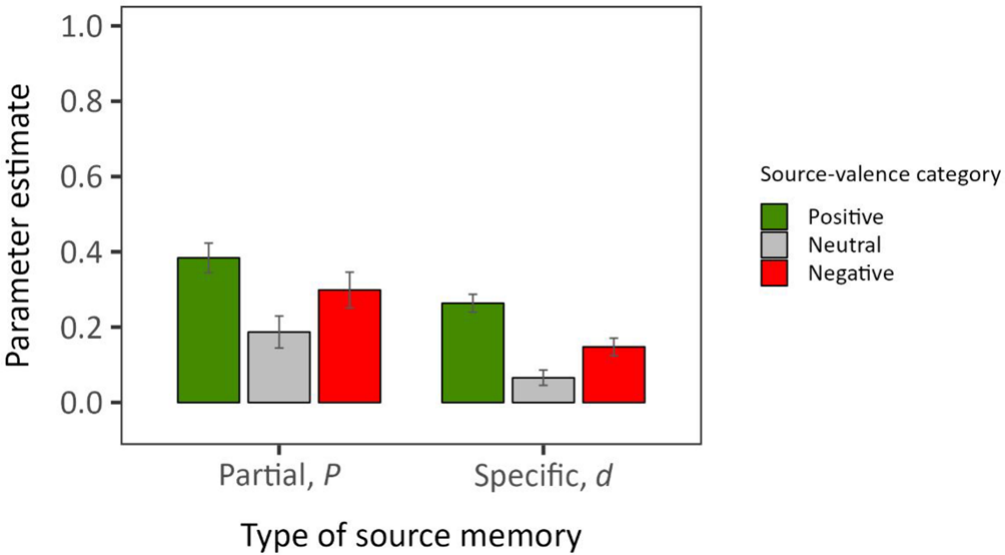
Depicted are partial and specific source memory, measured by parameter *P* and *d* of the Partial-Source Model, respectively (Dodson et al., 1998), as a function of the source-valence category (positive, neutral, negative). Error bars indicate one standard error of estimate. (Color figure online)

As evident in Fig. 3, partial source memory significantly differed across source-valence categories, *ΔG*^*2*^(2) = 11.29, *p* = .004. Follow-up pairwise comparisons showed that partial source memory for the positive source-valence category was higher than for the neutral one, *ΔG*^*2*^(1) = 11.20, *p* < .001. However, no significant difference emerged between negative and neutral partial source memory, Δ*G*^2^(1) = 2.52, *p* = .113, presumably due to lower power for detecting pairwise differences in *P* (compared with detecting an overall valence effect; for details on post hoc power analyses, see online supplement). Because positive and negative partial source memory also did not differ, Δ*G*^*2*^(1) = 1.80, *p* = .180, we equated the two parameters into a joint *P*_*emotional*_ parameter and compared it against the neutral parameter (*P*_*neut*_) for a more powerful test of emotion-enhanced effects (cf. Symeonidou & Kuhlmann, 2024). This yielded a significant difference, *ΔG*^*2*^(1) = 9.49, *p* = .002, suggesting that there was an overall emotion-enhanced benefit in partial source memory: Participants showed better source memory for the emotional compared to the neutral source categories.

#### Specific source memory

The specific source memory parameters also significantly differed across source-valence categories, Δ*G*^*2*^(2) = 40.10, *p* < .001 (see Fig. 3). Follow-up pairwise comparisons revealed better specific source memory when the source image was of positive, *ΔG*^*2*^(1) = 39.96, *p* < .001, or of negative valence, *ΔG*^*2*^(1) = 7.16, *p* = .007, compared with neutral valence. Further interestingly, specific source memory was significantly better for positive compared to negative source images, *ΔG*^*2*^(1) = 12.25, *p* < .001, implying a positivity effect.

## Discussion

The present study examined how source emotionality influences the specificity of source memory, and particularly whether an EEM effect occurs in both partial source memory (i.e., memory for the source-valence category), and specific source memory (i.e., memory for the specific source exemplar). To this end, we used neutral word items superimposed on one of three positive, three negative, or three neutral source images, that were similar within each valence category, and let participants rate the items in terms of pleasantness at encoding. At test, all nine source images were presented along with a new option, and participants provided a source judgment for previously learned and new items. The Partial-Source Model (Dodson et al., 1998; Klauer & Wegener, 1998) was applied to estimate guessing-corrected partial and specific source memory. Overall, the results provide evidence for an EEM effect for both partial and specific source memory. More specifically, for partial source memory, participants showed better source memory for the emotional compared with the neutral source categories. Notably, this effect was primarily driven by enhanced memory for the positive valence category, with participants remembering positive sources better than neutral ones, whereas the comparison between negative and neutral sources did not reach significance, presumably due to power limitations for small pairwise differences (see online supplement). For specific source memory, the results again revealed a robust EEM effect, with participants showing better memory for both positive and negative compared with neutral source images.

Our findings partly contrast with Bell, Buchner, et al. (2012), who did not observe EEM effects in specific source memory. While there are several procedural and material differences between their and our study, a key distinction lies in the retrieval demands: Bell, Buchner, et al. assessed specific source memory via recall, requiring effortful retrieval of source details, while partial memory was tested with a retrieval-facilitated forced-choice test. In contrast, in our study, retrieval demands were minimized for both partial and specific source memory because all source exemplars were presented at test (forced-choice test format). This facilitated access to specific source details and may explain why we found EEM effects in both partial and specific source memory.

Interestingly, for specific source memory, we additionally observed a positivity effect, with participants showing better specific source memory for positive compared to negative source images. A similar trend emerged in partial source memory, where the EEM effect was primarily driven by the positive valence category: the positive–neutral comparison was significant, whereas the negative–neutral difference was smaller and statistically unreliable. Nonetheless, the descriptive pattern for both partial and specific source memory was consistent, with performance highest for positive, followed by negative, and then neutral sources.

While this positivity effect contrasts with the well-established negativity bias in item memory (Baumeister et al., 2001), it aligns with previous findings on emotional sources. For instance, using a comparable encoding procedure, Symeonidou and colleagues repeatedly found higher source memory (more akin to partial source memory in their test design) for positive than negative (and neutral) sources across several experiments (Symeonidou & Kuhlmann, 2024; Symeonidou et al., 2022), with the positive-neutral difference being more reliably significant than the negative-neutral difference. Note, however, that some studies also report a negativity effect in emotional source memory (Bell & Buchner, 2012), but this seems specific for socially threatening sources.

Interestingly, Symeonidou and Kuhlmann (2024, Experiment 2) additionally showed that participants perceived a higher fit between neutral items and positive sources compared to negative and neutral sources, suggesting a higher relatedness for positive material. In a similar vein, Ventura-Bort et al. (2016) found that participants more successfully imagined neutral objects within positive versus negative or neutral contexts, again pointing towards greater relatedness. Given that source memory—in contrast to item memory—critically relies on the binding of item to source, this increased relatedness may have facilitated memory for positive sources. More broadly, there is evidence suggesting that positive stimuli generally exhibit greater associative potential than negative stimuli (Unkelbach et al., 2020). Relatedly, studies have found enhanced associative memory for positive versus negative or neutral word pairs (Madan et al., 2019). Future research could further explore whether the observed positivity effect in source memory is driven by greater associative potential, for example by additionally varying item valence and systematically assessing item-source relatedness (e.g., by collecting pre-ratings).

Despite several strengths of the present study, including the use of carefully selected, normed material, the application of MPT modeling, and the facilitation of retrieval during source testing, one important limitation should be acknowledged. Specifically, there is a potential confound between source valence and image category: For partial source memory, it is difficult to disentangle whether the observed memory benefits were driven by source valence or by specific characteristics of the image category itself. For instance, it is possible that the themes “lake” (positive) and “garbage” (negative) are inherently more memorable than the theme “car-race” (neutral), independent of their valence. However, this seems quite unlikely, especially given that the image categories were nearly matched on key perceptual features (entropy and SSIM), thus systematically differing primarily in their valence. A potential way to address this issue in future research would be to include multiple thematic categories within each valence condition (e.g., several distinct positive themes like lakes, sunsets, flowers). However, this approach would only shift the confound to specific source memory: memory for the specific source exemplar would be then confounded with memory for the thematic category. That is, partially remembering that a word was paired with a “lake” rather than a “sunset” would suffice to correctly identify the specific source, without recollecting specific visual details (see Bell , Giang, & Buchner, 2012, for a similar discussion). More broadly, this issue reflects a fundamental challenge in defining what constitutes the general source category versus specific source details and is thus not unique to the present study but applies to previous research on partial versus specific source memory as well. To illustrate, rather than criticizing the current study for using thematically similar source exemplars within a valence category, one could argue the opposite—namely, that the images within each category were *not* similar enough so that participants could rely on one single salient feature (e.g., a mountain in one of the lake images) to identify the correct specific source, without the need for detailed recollection of other image features. Future studies could attempt to systematically manipulate source specificity by varying the degree of shared versus unique features across exemplars within a category. However, increasing similarity between exemplars may also render the task overly difficult, potentially resulting in floor effects, particularly for neutral sources where specific source memory was already low in the current study.

Another more general limitation is the gender imbalance in the sample. Prior research indicates that gender can modulate emotional reactivity (Kurdi et al., 2017; Wrase et al., 2003). Indeed, for the images used in this study, the normative OASIS ratings showed a tendency toward stronger emotional responses to the positive and negative (but not the neutral) images among female raters (i.e., higher ratings of arousal and absolute valence). It is therefore possible that the observed EEM effects would be weaker in a more gender-balanced sample. Future research should employ more gender-balanced and, more generally, more diverse samples to assess the robustness of these effects.

Notwithstanding these limitations, the current experimental design successfully assessed source memory at two different levels of specificity and allows concluding that source emotionality influences source memory across these levels.

## Supplementary Information

Below is the link to the electronic supplementary material.

Supplementary file1 (DOCX 146 kb)

## Data Availability

The datasets generated and analyzed for the current study are available in the Open Science Framework (OSF) repository (https://osf.io/brpg7/). The word list used in the study is also available there. For the used source images, we provide the original file names in Table 1. The images can be obtained from the link provided in the original OASIS paper (Kurdi et al., 2017). The experiment was not preregistered.

## Footnotes

1 It is crucial to distinguish this line of research from studies examining the more extensively studied effects of emotional items on source memory for neutral contextual details (see Pereira et al., 2023, for a meta-analysis).

2 To ensure that the extension to three sources resulted in a mathematically identifiable model, we ran identifiability checks using the respective built-in function in the software multitree (Moshagen, 2010). More specifically, identifiability is assessed by imputing random parameter values into the model, generating response frequencies based on these values, reestimating the parameters from the frequencies, and comparing the estimates to the originally imputed values to evaluate deviations. This was done for 200 simulated data sets (i.e., 200 × 30 parameters = 6,000 possible cases of deviation). In only 0.23% of all cases, a very small, negligible deviation (of maximum 0.01) between original and estimated parameter values emerged, supporting the global identifiability of the extended model version.

## Code availability

The Google Colab script used to calculate entropy and SSIM values for the selected source-images can be found online (https://colab.research.google.com/drive/1ry8Wksk2ACYE1Q-dP3vg8KaFlThhDZox?usp=sharing). The code supporting the ANOVA results reported in this manuscript is also available at the above provided OSF link. The model-based analysis was conducted with the GUI-based program mutiTree (Moshagen, 2010) and thus no code can be generated. However, a detailed output of all performed model-based analyses was uploaded under the same OSF link.
