# Supplementary material for: Emotional valence boosts partial and specific source memory
Source: Psychon Bull Rev. 2026 Apr 2;33(4):129. doi: 10.3758/s13423-026-02888-6 (PMC13046597; doi:10.3758/s13423-026-02888-6)
Supplement: Supplementary file 1 — Supplementary file1 (DOCX 146 kb) [file 13423_2026_2888_MOESM1_ESM.docx]

**Online Supplement**

to

**Emotional Valence Boosts Partial and Specific Source Memory**

Nikoletta Symeonidou*, Maria Lee, & Beatrice G. Kuhlmann

*Department of Psychology, University of Mannheim, Germany*

**Author Note**

Nikoletta Symeonidou [
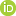
](https://orcid.org/0000-0003-1852-5019) <https://orcid.org/0000-0002-2471-8653>

Maria Lee, Department of Psychology, School of Social Sciences, University of Mannheim.

Beatrice G. Kuhlmann [
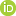
](https://orcid.org/0000-0003-1852-5019) <https://orcid.org/0000-0002-3235-5717>

*Correspondence concerning this article should be addressed to Nikoletta Symeonidou, Department of Psychology, University of Mannheim, D-68161 Mannheim, Germany. +49(0)621-181-3390, E-mail: [nsymeoni@mail.uni-mannheim.de](mailto:nsymeoni@mail.uni-mannheim.de).

# **Supplemental Results**

**Table S1**

*Estimates of all Parameters of the Partial-Source Model*

| Process | | Parameter name | Estimate | 95% confidence intervals |
| --- | --- | --- | --- | --- |
| Probability of **recognizing an item** as previously presented or not presented | ***D*** | .75 | [.72; .77] |  |
| Probability of guessing that an item is old | *b* | .44 | [.39; .48] |  |
| Probability of identifying a specific negative source exemplar **(specific source memory)** | ***d_neg_*** | .15 | [.10; .19] |  |
| Probability of identifying a specific neutral source exemplar **(specific source memory)** | ***d_neut_*** | .07 | [.03; .11] |  |
| Probability of identifying a specific positive source exemplar **(specific source memory)** | ***d_pos_*** | .26 | [.22; .31] |  |
| Probability of identifying the negative valence category (**partial source memory)** | ***P_neg_*** | .30 | [.21; .39] |  |
| Probability of identifying the neutral valence category (**partial source memory)** | ***P_neut_*** | .19 | [.10; .27] |  |
| Probability of identifying the positive valence category (**partial source memory)** | ***P_pos_*** | .38 | [.31; 46] |  |
| Probability of guessing the positive source-valence category for recognized words | *a_pos_* | .29 | [.25; .33] |  |
| Probability of guessing the negative source-valence category for recognized words | *a_neg_* | .57 | [.51; .63] |  |
| Probability of guessing the positive source exemplar 1 for recognized words | *a_pos_1_* | .37 | [.31; .42] |  |
| Probability of guessing the positive source exemplar 2 for recognized words | *a_pos_2_* | .59 | [.52; .65] |  |
| Probability of guessing the negative source exemplar 1 for recognized words | *a_neg_1_* | .28 | [.23; .32] |  |
| Probability of guessing the negative source exemplar 2 for recognized words | *a_neg_2_* | .59 | [.53; .64] |  |
| Probability of guessing the neutral source exemplar 1 for recognized words | *a_neut_1_* | .41 | [.36; .47] |  |
| Probability of guessing the neutral source exemplar 2 for recognized words | *a_neut_2_* | .50 | [.42; .58] |  |
| Probability of guessing the positive source-valence category for *un*recognized words | *g_pos_* | .31 | [.22; .39] |  |
| Probability of guessing the negative source-valence category for *un*recognized words | *g_neg_* | .44 | [.33; .56] |  |
| Probability of guessing the positive source exemplar 1 for *un*recognized words | *g_pos_1_* | .49 | [.33; .66] |  |
| Probability of guessing the positive source exemplar 2 for *un*recognized words | *g_pos_2_* | .47 | [.24; .71] |  |
| Probability of guessing the negative source exemplar 1 for *un*recognized words | *g_neg_1_* | .39 | [.23; .56] |  |
| Probability of guessing the negative source exemplar 2 for *un*recognized words | *g_neg_2_* | .50 | [.29; .70] |  |
| Probability of guessing the neutral source exemplar 1 for *un*recognized words | *g_neut_1_* | .25 | [.12; .37] |  |
| Probability of guessing the neutral source exemplar 2 for *un*recognized words | *g_neut_2_* | .49 | [.32; .65] |  |

*Note.* All memory parameters are in bold.

# **Supplemental Analysis 1: Post-hoc Power Analysis**

## **General Valence Effects**

As noted in the main text, there was no adequate prior study (using similar material, paradigm, and model) to inform an a priori power analysis. We therefore based our sample size on Experiment 1 of Symeonidou and Kuhlmann (2024), who used a similar procedure and found a reliable source-memory benefit for emotional source categories with *N* = 68. Although, admittedly, this study did not differentiate between partial and specific source memory, the therein reported emotionality effect in source memory is akin to an emotion-based benefit in partial source memory (i.e., source memory for the valence category). Crucially, we additionally conducted a post-hoc power analysis to assess whether our sample size of N = 67 was sufficient to detect parameter differences of .15 in both partial and specific source memory as a function of source valence. The assumed difference of .15 represented a conservative estimate drawing on the results in Symeonidou and Kuhlmann (2024), where valence-related effects in source memory ranged from .16 (Experiment 2, affective condition) to .29 (Experiment 1).

To conduct this analysis, we used the software *multiTree* (Moshagen, 2010) and its built-in function for post-hoc power analyses. More specifically, all empirically observed parameter estimates (see Table 2 of the main paper) entered the power analysis with the following exceptions: For partial source memory, the parameters P_pos_ and P_neg_ were assumed to be .34, based on the observed value of P_neut_ = .19 plus the .15 difference (i.e., .19 + .15 = .34). For specific source memory, d_pos_ and d_neg_ were set at .22, based on the observed d_neut_ value of .07 (i.e., 0.7 + .15 = .22). Assuming α = .05, the analysis yielded that for detecting a general valence effect (i.e., by comparing emotional sources against the neutral source) statistical power was 1-β = .81 for partial source memory (P_pos_ = P_neg_ ≠ P_neut_), and 1-β = .99 for the specific source memory (d_pos_ = d_neg_ ≠ d_neut_), respectively. Thus, with *N* = 67, the study had sufficiently high power to detect small valence effects (i.e., .15 parameter difference) in both partial and specific source memory parameters.

## **Pairwise Comparisons**

In contrast, for the p**airwise comparisons, statistical power was** considerably **lower. The same parameter values entered this power analysis as described above with the crucial difference that we now looked at pairwise differences (e.g.,** P_neg_ ≠ P_neut_**; and** P_pos_ ≠ P_neut_) **instead of testing for the presence of a general valence effect (see above). For partial source memory, this analysis yielded a power of** 1-β = .71 for the positive-neutral, and 1-β = .59, for the negative-neutral comparison for detecting the assumed difference of .15. Thus, the study was not sensitive enough to detect differences of smaller than .15, as observed in our data. This can explain why the negative-neutral difference for partial source memory (difference of .11) was descriptively present but not significant.

Notably, for specific source memory, the power was 1-β = .99 for both pairwise comparisons (positive-neutral and negative-neutral). This results from the Partial-Source Model structure: partial source memory is estimated only when specific source memory fails and therefore relies on fewer data points (see model illustration in Figure 2 in the main manuscript). In contrast, the estimation of the specific source memory parameter is based on more data points, yielding higher estimation precision – and thus greater statistical power – for the same sample size, α level, and parameter difference

Taken together, our sample size was sufficient to detect small pairwise differences in specific source memory. However, power was lower for detecting pairwise differences in partial source memory, particularly between negative and neutral conditions, suggesting that this non-significant difference may be due to limited statistical power rather than the absence of a true effect (see main text).

# **Supplemental Analysis 2: Analysis of Source Accuracy**

### **Accuracy for Source-Valence Category**

A prevalent performance measure for source memory is the conditional source identification measure (CSIM; Murnane & Bayen, 1996). The CSIM represents the proportion of correct item attributions to a specific source among all correctly recognized items (i.e., hits) from that source (see Table S2-1 for all response frequencies per source-valence category). Thus, in our case, the CSIM scores for the negative, neutral, and positive source categories reflect the proportion of correct item attributions to the respective valence category among all correctly recognized items of that valence category.

Notably, however, unlike the model-based approach used in our main analyses (i.e., Partial-Source Model, Dodson et al., 1998; Klauer & Wegener, 1998), CSIMs do not account for systematic guessing biases and may therefore yield biased estimates and potentially misleading conclusions. Their advantage is that they can be analyzed using standard statistical methods (e.g., analysis of variance), but results should be interpreted with caution. To assess whether and how result patterns varied depending on the analysis method, we ran a within-participants ANOVA with CSIMs as dependent variable and the source-valence category (positive, neutral, negative) as independent variable. Results indicated a main effect of the source-valence category, *F*(1.76, 115.80) = 24.10, *p* < .001, η²_p_ = .27 (Greenhouse-Geisser corrected). Bonferroni-Holm adjusted pairwise comparisons further showed that source accuracy was better for the positive and negative compared to the neutral source category, *t*(66) = 5.69, *p* < .001, *d* = 0.77, and *t*(66) = 5.31, *p* < .001, *d* = 0.69, with no difference between negative and positive categories, *t* < 1. This suggests an emotionality effect in participants’ accuracy for both the positive and negative source-valence category. This only in part replicates MPT results where only the positive-neutral pairwise comparison was significant but note that the descriptive pattern (illustrated in Figure S2-1) was consistent across analysis methods. This deviation can be reconciled when considering the guessing parameter estimates presented in Table S1 above: There was a bias towards guessing the negative source valence category for recognized items (i.e., *a_neg_* > .50), whereas the positive category was guessed at chance level. That is, CSIM for the negative source valence was inflated by this guessing bias, rendering it closer to the positive CSIM than the guessing-corrected source memory parameters were.

**Table S2-1**

*Response Frequencies per Source-Valence Category*

|  | Valence response | | | |
| --- | --- | --- | --- | --- |
| Correct source-valence category | Lake  (positive) | Car-race (neutral) | Garbage  (negative) | New |
| Lake (positive) | **558** | 151 | 179 | 117 |
| Car-race (neutral) | 193 | **387** | 258 | 167 |
| Garbage (negative) | 162 | 181 | **517** | 145 |
| New | 34 | 42 | 33 | **896** |

*Note.* Frequencies were based on the valence category only, disregarding the specific source image.

**Accuracy for Specific Source Image**

**Figure S2-1**
Source Identification for the Source-Valence Category


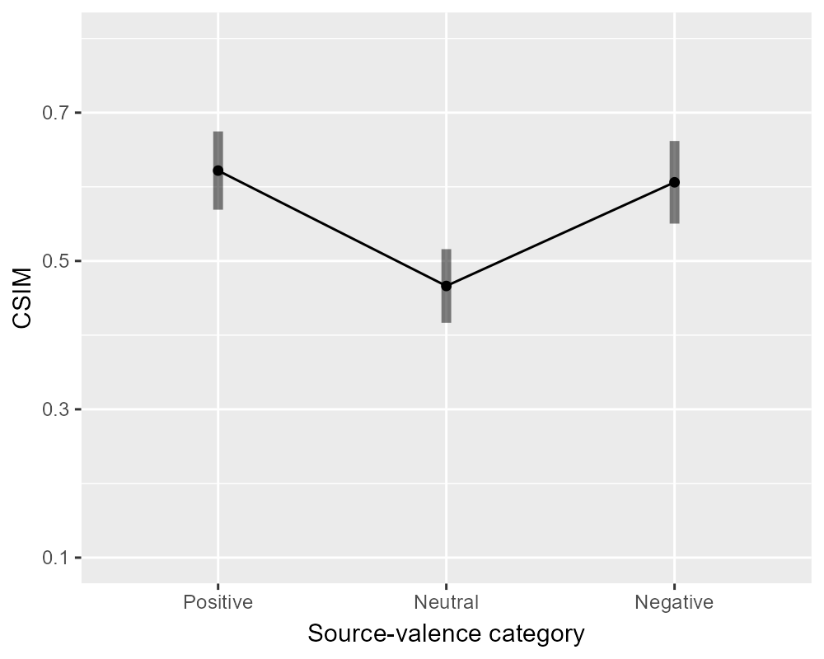


Note. The figure shows conditional source identification performance (CSIM) for the source category as a function of its valence (positive, neutral, negative). Error bars represent 95% confidence intervalls.

Accordingly, to investigate accuracy for the specific source image, we first calculated CSIMs for each specific image of a category (i.e., proportion of correct attributions to a specific source image among all category-consistent source responses). For example, the CSIM score for the *Car-Race-1* image denotes the proportion of correct *Car-Race-1* identifications among all *Car-Race* responses for items originally paired with that image. We then averaged across CSIMs of the three images of the same valence category – assuming that source accuracy did not differ for exemplars of the same valence category (see main paper) – which resulted into an average CSIM score (ACSIM; Murnane & Bayen, 1996) for each valence category. Put simply, these ACSIM scores capture specific source accuracy separately for each valence category. We then submitted these scores to a within-participants ANOVA with source-valence category (positive, neutral, negative) as independent variable. Results indicated, a main effect of the source-valence category, *F*(2.00, 131.69) = 7.89, *p* < .001, η²_p_ = .11. Bonferroni-Holm adjusted pairwise comparisons further showed that specific source accuracy was better for the positive compared to the neutral valence category, *t*(66) = 3.871, *p* < .001, *d* = 0.48. There was also a descriptive, however non-significant, trend towards higher specific source accuracy for negative compared to neutral valence, *t*(66) = 1.747, *p* = .085, *d* = 0.21, and for the positive compared to negative valence, *t*(66) = 2.258 , *p* = .055, *d* = 0.274. Thus, the results were not fully in line with the model-based analysis, where we found better specific source memory for both positive and negative compared to neutral source valence and an additional positivity bias (positive > negative). Note, however, that the descriptive pattern (see Figure S2-2) again replicated across analysis methods. Result differences might be in part attributed to the fact that such accuracy measures to not account for systematic guessing biases, which revealed guessing biases for some of the emotional images (see Table S1).


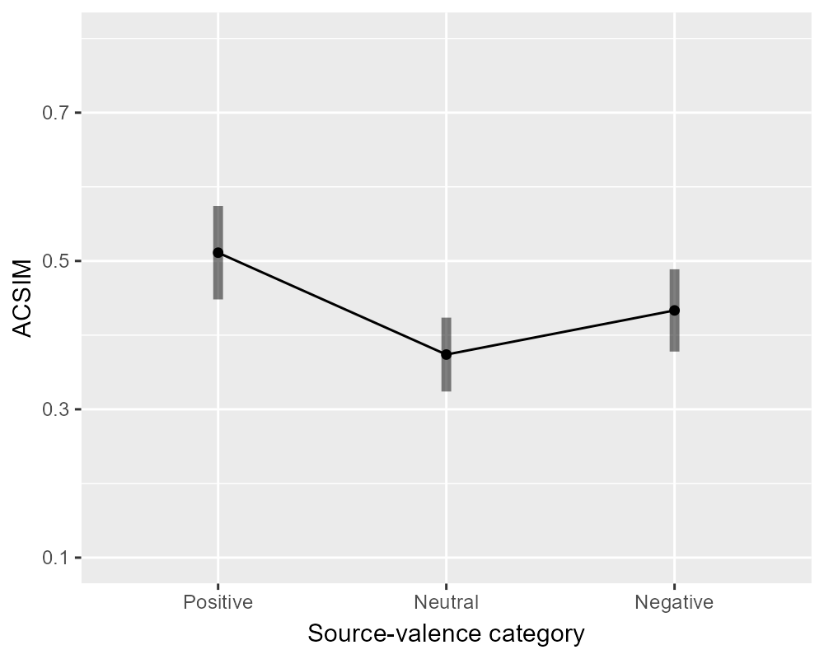


**Figure S2-2**
Source Identification for the Specific Source Image

Note. The figure shows the average conditional source identification performance (ACSIM) for the specific source image as a function of its valence (positive, neutral, negative). Error bars represent 95% confidence intervalls.

# **References**

Dodson, C. S., Holland, P. W., & Shimamura, A. P. (1998). On the recollection of specific- and partial-source information. *Journal of Experimental Psychology: Learning, Memory, and Cognition*, *24*(5), 1121–1136. https://doi.org/10.1037/0278-7393.24.5.1121

Klauer, K. C., & Wegener, I. (1998). Unraveling social categorization in the “Who said what?” paradigm. *Journal of Personality and Social Psychology*, *75*(5), 1155–1178. https://doi.org/10.1037/0022-3514.75.5.1155

Moshagen, M. (2010). MultiTree: A computer program for the analysis of multinomial processing tree models. *Behavior Research Methods*, *42*(1), 42–54. https://doi.org/10.3758/BRM.42.1.42

Murnane, K., & Bayen, U. J. (1996). An evaluation of empirical measures of source identification. *Memory & Cognition*, *24*(4), 417–428. https://doi.org/10.3758/BF03200931

Symeonidou, N., & Kuhlmann, B. G. (2024). Enhanced source memory for emotionally valenced sources: Does an affective orienting task make the difference? *Cognition and Emotion*, 1–22. https://doi.org/10.1080/02699931.2024.2309707
